# Supplementary material for: Thermodynamic evidence for a dual transport mechanism in a POT peptide transporter
Source: eLife. 2014 Dec 2;3:e04273. doi: 10.7554/eLife.04273 (PMC4271188; doi:10.7554/eLife.04273)
Supplement: Figure 3—source data 1. — DOI: http://dx.doi.org/10.7554/eLife.04273.010 [file elife04273s001.pdf]

Figure 3 – source data 1

pH In	pH Out	n/m (stoichiometry, proton:peptide)	Peptide in (mM)	Peptide out (mM)	Delta psi*
6.8	6.8	1	10	0.1	-120
6.8	6.8	2	10	0.1	-60
6.8	6.8	3	10	0.1	-40
6.8	6.8	4	10	0.1	-30
6.8	6.8	5	10	0.1	-24
6.8	6.8	6	10	0.1	-20
6.8	6.0	1	10	0.1	-72
6.8	6.0	2	10	0.1	-12
6.8	6.0	3	10	0.1	8
6.8	6.0	4	10	0.1	18
6.8	6.0	5	10	0.1	24
6.8	6.0	6	10	0.1	28
